# Unlocking Terpenoid Transformations: C–H Bond Functionalization for Methyl Group Substitution, Elimination, and Integration

**DOI:** 10.1021/acscentsci.4c01569

**Published:** 2024-10-11

**Authors:** Lauren A. M. Murray

**Affiliations:** Department of Biochemistry, Biomedicine Discovery Institute, Monash University, Clayton, Victoria 3800, Australia

Terpenoids are one of the largest classes of natural products, encompassing a diverse group of molecules with a wide variety of biological activities.^1^ These molecules are biosynthesized through the cyclization of polyisoprene precursors, followed by downstream modifications with tailoring enzymes. Given their structural diversity and abundance in nature, it is no surprise that many terpenoids and their derivatives have been investigated and exploited for their therapeutic potential. The abundance of sp^3^-hybridized carbon atoms, in the form of methyl groups (CH_3_), within many of these terpenoid molecules enable them to bind biological targets with high specificity, and can also play key roles in their conformation and solubility. Despite the widespread presence and functional importance of methyl groups within these scaffolds, methods to selectively transform these groups in such structures are limited. Thus, the development of efficient strategies to further functionalize these molecules continues to be of interest and has significant implications in the development of terpenoid-based pharmaceuticals.

**Figure 1:**
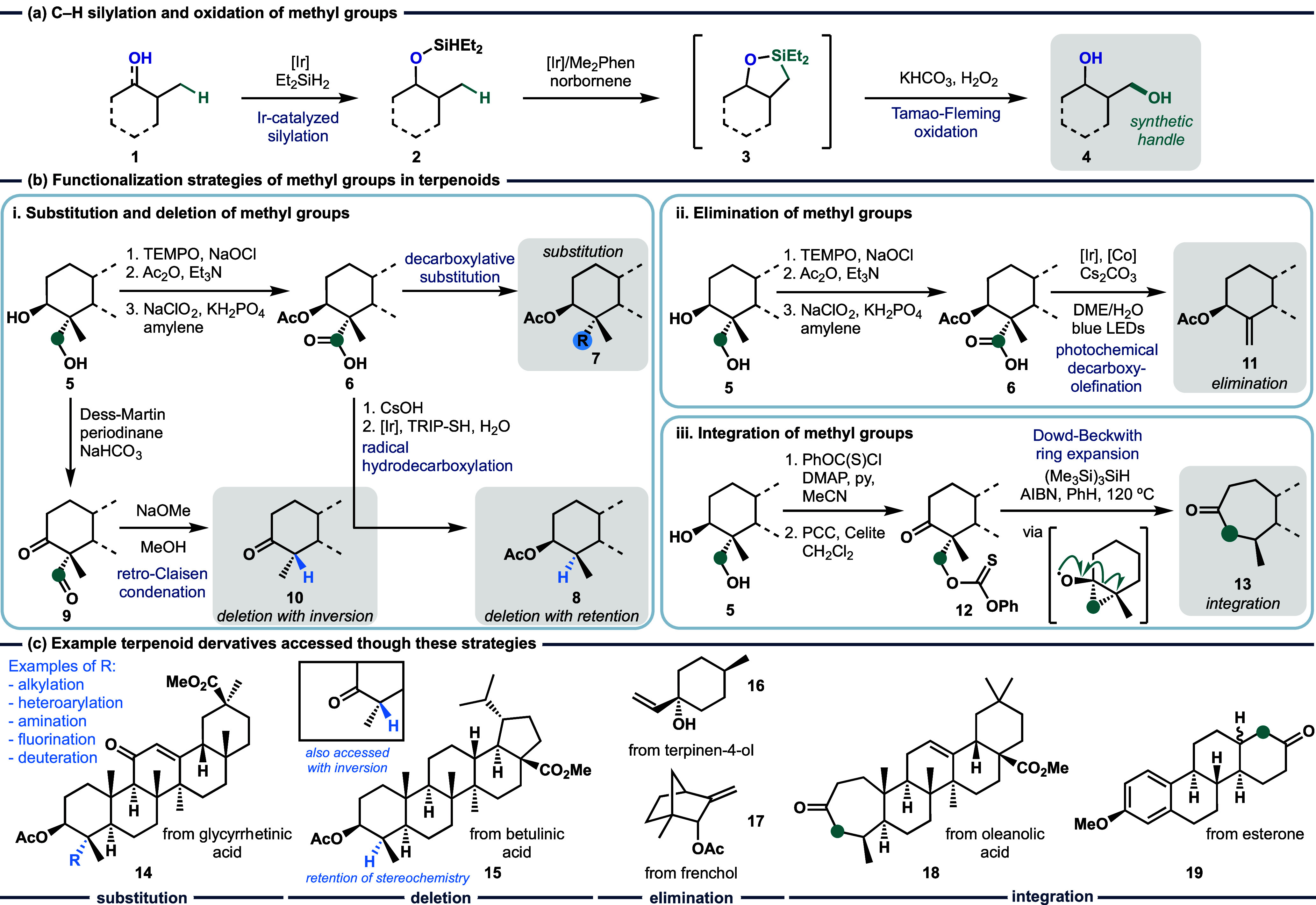
(a) C–H silylation and oxidation of terpenoids directed by alcohols or ketones. (b) Functionalization strategies of methyl groups in terpenoids. **i.** Substitution and deletion of methyl groups. **ii.** Elimination of methyl groups. **iii.** Integration of methyl groups. (c) Example terpenoid derivatives accessed through these strategies.

In this issue of *ACS Central Science*, Hartwig and co-workers describe the site-selective functionalization of methyl groups in a range of terpenoid natural products.^2^ Through a sequence of C–H silylation and oxidation, the newly installed hydroxyl group serves as a synthetic handle for subsequent substitution, elimination, or integration of the methyl carbon into the terpenoid skeleton by the cleavage of C–C bonds (Figure 1). While nature has evolved elegant strategies to functionalize C–H bonds to initiate C–C bond cleavage, synthetic methods toward this goal commonly require the preinstallation and subsequent removal of specific directing groups to control the site of functionalization, adding additional steps to a synthetic route. As outlined below, the synthetic approach described by Hartwig and co-workers overcomes this downfall by strategically utilizing the natural abundance of hydroxyl and carbonyl groups in terpenoid natural products as native directing groups for an iridium-catalyzed C(sp^3^)–H silylation and oxidation sequence. This hydroxymethyl moiety is then carried forward into synthetic sequences that lead to functionalization of the methyl group on a range of complex terpenoid scaffolds.

Building on previous work,^3^ the authors have extended their efficient strategy for methyl group C–H functionalization through an iridium-catalyzed C–H silylation and oxidation sequence (Figure 1a). This reaction sequence was successfully carried out on a variety of monoterpenoids, sesquiterpenoids, triterpenoids, and steroids, producing the desired diol that would allow for subsequent functionalization of the methyl group.

Following successful methyl group hydroxylation on a series of terpenoids, the authors investigated various methods for substitution, elimination, and integration of the hydroxymethyl group (Figure 1b). A formal substitution of the methyl group would allow a classically challenging late-stage diversification of the terpenoid skeleton, converting the unreactive methyl to a range of functional handles (Figure 1bi). Such substitutions were achieved by converting the newly installed primary alcohol (**5**) to a carboxylic acid and protecting the natural secondary alcohol as an acetate (**6**). The newly furnished carboxylic acid moiety then underwent substitution through various photocatalytic decarboxylations, leading to the formal substitution of the methyl group in **7** with a series of alkyl, aryl, and amine groups, as well as substitution with fluorine and deuterium. The exchange of such methyl groups for heteroaryl and amine moieties has the potential to improve binding and activity of these scaffolds, as seen in hundreds of FDA-approved drugs containing nitrogen-based heteroarenes, amino groups, or amide derivatives.^4^

This work also extended substitution chemistry to the formal substitution of the methyl group with hydrogen, resulting in a methyl group deletion. This deletion is essential in the biosynthesis of cholesterol, as well as plant and fungal sterols, and is known to significantly impact the binding affinities of drugs. In this case, following the synthesis of the intermediate acid (**6**), decarboxylation was performed under photocatalytic conditions to afford the corresponding demethylated terpenoids (**8**) in high yields with complete retention of stereochemistry. This procedure elegantly avoided preinstallation of a thiohydroxamate ester, as required in a more conventional Barton decarboxylation. The authors also achieved demethylation with net inversion of stereochemistry, taking advantage of a sodium methoxide mediated retro-Claisen condensation of an intermediate aldehyde (**9**), leading to a loss of the methyl group as sodium formate to afford **10**.

Following substitution chemistry, the authors set out to excise the functionalized methyl group, while simultaneously forming an olefin through a formal elimination of methane (Figure 1bii). This newly formed alkene moiety would allow for further downstream modification of the terpenoid skeleton. This procedure followed the same sequence of oxidation to the carboxylic acid (**6**), followed by a photochemical decarboxyolefination protocol reported by Ritter^5^ to form an alkene (see **11**) in place of a *gem*-dimethyl unit. Notably, the authors observed complete selectivity for the terminal alkene, even in cases where the internal isomer could form.

Finally, the hydroxylated methyl group could also be integrated into the ring system of the terpenoid (Figure 1biii). This allowed for a one carbon ring-expansion of the carbon skeleton, producing rare or unknown scaffolds with the potential for unique physicochemical or biological properties. Terpenoid ring expansions are typically accessed through a three-step sequence, implementing a Tiffeneau-Demjanov rearrangement that is often unselective due to the migration of either alkyl substituent, resulting in a mixture of constitutional isomers. This was overcome by implementing a Dowd-Beckwith-type ring expansion using a thionocarbonate intermediate. First, the newly installed hydroxymethyl group of **5** was selectively converted to the corresponding thionocarbonate, followed by oxidation of the remaining hydroxyl group with pyridinium chlorochromate (PCC) to afford ketone **12**. Next, a well-designed interception of the primary radical formed under Barton–McCombie deoxygenation conditions allowed formation of a cyclopropane intermediate (shown) via addition to the ketone. The cyclopropane then opened to form a tertiary alkyl radical, which, following hydrogen atom abstraction, furnished the ring-expanded product **13**.

These three strategies of substitution, elimination, and integration allowed the authors to access a range of terpenoid derivatives (Figure 1c), with this work describing the evaluation of over 30 distinct scaffolds. This approach allows access to selectively functionalized complex terpenoid frameworks, many of which have been investigated for their therapeutic activity. Notably, the authors further proved the synthetic utility of this work by accessing glycyrrhetinic acid derivatives (see **14**), some of which are drug candidates for the treatment of hyperkalemia, improving on previous syntheses in both yield and step count.

Overall, this report offers a wealth of methods for selective functionalization of terpenoid methyl groups, enabling deeper exploration of their therapeutic potential and mechanisms. The synthetic diversity achieved through C–H activation of the terpenoid methyl group underscores the exciting potential of this methodology to reignite the development of previously overlooked compounds, and to access previously inaccessible new-to-nature molecules with unexplored properties. Not only will this strategy have applications in the synthesis and pharmacology of terpenoid derivatives, but its broader implications will allow for the functionalization of other natural products and synthetic intermediates bearing methyl groups.
